# Evaluation of Matrix-assisted laser desorption/ionization Time-of flight Mass spectrometry (MALDI TOF MS) and VITEK 2 in routine microbial identification

**DOI:** 10.4314/gmj.v55i4.12

**Published:** 2021-12

**Authors:** Anitha Madhavan, Arun Sachu, Nandini Sethuraman, Anil Kumar, Jayalakshmi Vasudevapanicker

**Affiliations:** 1 Department of Microbiology, Government TD Medical College, Alappuzha, Kerala, India; 2 Department of Microbiology, Believers Church Medical College, Thiruvalla, Kerala, India; 3 Department of Microbiology, Apollo Hospital, Chennai, India; 4 Department of Microbiology, Amrita Institute of Medical Sciences and Research Centre, Kerala, India

**Keywords:** MALDI TOF MS, VITEK -2, Sequencing

## Abstract

**Bacground:**

Microbial Identification was done by phenotypic methods. VITEK-2 and Matrix-assisted laser desorption ionization-time of flight mass spectrometry (MALDI-TOF MS) are now being increasingly used in laboratories.

**Objectives:**

To compare and evaluate the usefulness of MALDI-TOF MS and VITEK-2 in routine microbial identification.

**Methods:**

The performances of MALDI-TOF MS and VITEK 2 were compared for identifying microorganisms.

**Results:**

MALDI- TOF MS and VITEK-2 correctly identified 96 % (96/100) and 97% (97/100) of the isolates upto the genus level.

**Conclusion:**

MALDI TOF MS and VITEK -2 gave comparable identification and error rates. The rapid reduction in turnaround time with MALDI TOF is a significant game-changer in the field of clinical microbiology.

**Funding:**

State Board of Medical Research (SBMR).

## Introduction

Microbial identification in laboratories is achieved mainly by observing colony morphology, grams stain and biochemical reactions.^1^ A laboratory usually takes 48–72 hrs after receiving the sample to identify the organism.^2^ Vitek 2 System (Biomerieux-Vitek) is one of the automated systems which can identify gram-negative bacteria (GNB) within three hours.^3^ VITEK 2 can accurately identify 84.7% of the gram-negative bacteria within three hrs as shown by Funke et al .^4^ MALDI-TOF MS technology has a simple, high throughput, and low-cost technique plus a larger database and a more rapid turnaround time (few minutes).^5^ The prime objective was to use two systems to identify 100 isolates.

## Methods

This was a study conducted in Government Medical College, Alleppey from September 2018 to February 2019. Isolates(n=100) such as those belonging to Enterobacteriaceae, non-fermenting GNB, yeasts were used. This study was approved by the Institutional Ethics Committee (Reg.No. ECR/122/ Inst/KL/2013/RR-16) of Government Medical College, Alleppey (Reference Number-EC 38/2018).

VITEK used Gram-negative (GN), Gram-positive (GP) and Yeast cards for identification. An identification rate greater than 90% was considered acceptable. For MALDI-TOF MS, Isolate was smeared on the target slide with a wooden stick and was then smeared with 1 µL VITEK MS-CHCA (α-Cyano-4-hydroxycinnamic acid) and air-dried until the matrix and sample co-crystallized. In the case of yeast,1.5 µL of 70% formic acid was added to each isolate and dried. Then, 1.5 µL of an α-cyano-4-hydroxycinnamic acid (CHCA) matrix solution was added and dried again. The mass spectra acquired for each sample were compared to the reference spectra. *16S rRNA* gene sequencing was used to resolve all genus level discrepancies.

### Errors and Identification standards

Concordant - If the isolates were correctly identified at the genus or species level by both systems.

Major Errors -Discordant results at the genus level or if the results from one method were not definite.

Minor Errors- Discordant results at the species level but not at the genus level.

## Results

MALDI- TOF MS and VITEK-2 correctly identified 96 %(96/100) and 97% (97/100) of the isolates up to the genus level. The identification rates for MALDI- TOF MS and VITEK-2 up to species level were 92.9% (92/99) and 90.9% (90/99) respectively (One Minor error was not taken for calculation up to species level as Sequencing was not done for this isolate). Among the 100 isolates, results for 93 isolates were in agreement up to genus level by both methods. Results for 87 isolates were in agreement up to species level by both methods (Table-1). So among the 100 isolates, 13 (13%) isolates produced discordant results between the two methods.

**Table 1:** Matching results by both MALDI TOF and VITEK

Identification	Number of Strains n (%)
**Acinetobacter baumanii**	16 (18.4)
**Acinetobacter nosocomialis**	1 (1.1)
**Burkholderia cepacia**	16 (18.4)
**Citrobacter freundii**	2 (2.3)
**Candida albicans**	3 (3.4)
**Candida tropicalis**	7 (8)
**Candida parapsilosis**	2 (2.3)
**Enterobacter cloacae**	4 (4.6)
**Escherichia coli**	14 (16.1)
**Klebsiella pneumonia**	2 (2.3)
**Kodamaea ohmeri**	1 (1.1)
**Pseudomonas aeruginosa**	2 (2.3)
**Pseudomonas putida**	2 (2.3)
**Pseudomonas stutzeri**	1 (1.1)
**Salmonella group**	4 (4.6)
**Serratia marcescens**	1 (1.1)
**Shewanella putrefaciens**	1 (1.1)
**Staphylococcus haemolyticus**	1 (1.1)
**Staphylococcus saprophyticus**	2 (2.3)
**Stenotrophomonas maltophilia**	3 (3.4)
**Trichosporon asahii**	2 (2.3)

### Major Errors

Among the seven isolates which were not in agreement at the genus level, two organisms were identified as *Shigella sonnei* and Escherichia coli by VITEK and MALDI-TOF MS respectively. Isolates were confirmed as *Shigella sonnei* by serotyping. These isolates were not processed as *16S rRNA* gene sequencing should not be used to differentiate between *E. coli* and shigella.^6^

So, by excluding these two organisms, there were five major errors (Table 2). These were resolved by sequencing. VITEK and MALDI TOF MS showed 3% and 2% errors respectively.

**Table 2:** Major Errors

VITEK	MALDI TOF	Sequencing
**Sphingomonas paucimobilis**	Brevibacillus	Brevibacillus agri
**Sphingomonas paucimobilis**	Delftia acidovorans	Delftia tsuruhatensis
**Cupriavidus pauculus**	Acinetobacter baumanii	Acinetobacter baumanii
**Enterobacter cloacae**	Leclercia adecarboxylata	Enterobacter cloacae
**Candida ciferii**	Not identified	C.allociferii

### Minor Errors

There were six isolates in our study for which there was agreement upto the genus level but there was discordance at species level. Among the three Candida species, two were identified as *C. parapsilosis* and *C.orthopsilosis* by VITEK and MALDI-TOF MS respectively. This is due to the fact that *Candida orthopsilosis* is not present in the database of VITEK. Among the three bacterial isolates, two were identified as *Elizabethkingia anopheles* and *Citrobacter werkmanii* by MALDI TOF MS and the corresponding VITEK identifications were *E. meningospeptica* and *C.freundii* respectively. This is due to the fact that *E. anopheles* and *C. werkmanii* are not present in the database of VITEK. So by excluding these organisms there were two minor errors, one of which included wrong identification of *C.albicans* as *C. lusitaniae* by VITEK-2 which was resolved by sequencing. The other minor error was the identification of an organism as *Acinetobacter junii* and *Acinetobacter baumanii* by VITEK-2 and MALDI-TOF respectively. *16S rRNA* gene sequencing was used to resolve only one of the minor errors due to financial limitations.

## Discussion

VITEK-2 showed identification rates of 97% and 90.9% at genus and species level whereas MALDI TOF MS showed identification rates of 96% and 92.9% respectively at genus and species level. Guo et al reported identification rates of 99.60% and 93.37 % up to genus and species level by MALDI TOF .^7^ Our findings were comparable to study by Van Veen *et al.*, who achieved identification rates of 97.1% and 92% up to genus and species level.^8^ This difference is due to the different choices of strains.

One major issue which was seen during the usage of MALDI TOF was the inability to differentiate between E. coli and shigella which has been previously documented .^9^ *gyrB* analysis of bacteria as an alternative to differentiate closely related species was mentioned by Fukushima et al .^10^ Another method for differentiating E.coli from Shigella was by using MALDI-TOF MS-based assay using ClinProTools software .^11^

## Conclusion

The results of this study showed that MALDI TOF MS and VITEK -2 gave comparable identification and error rates. However, the rapid reduction in turnaround time with MALDI TOF is a significant game changer in the field of clinical microbiology.
